# Pyrvinium pamoate regulates MGMT expression through suppressing the Wnt/β-catenin signaling pathway to enhance the glioblastoma sensitivity to temozolomide

**DOI:** 10.1038/s41420-021-00654-2

**Published:** 2021-10-12

**Authors:** Haisong Li, Shuhan Liu, Rihua Jin, Haiyang Xu, Yunqian Li, Yong Chen, Gang Zhao

**Affiliations:** 1 Department of Neurosurgery, The First Hospital of Jilin University, 130061 Changchun, China; 2 Cancer Center, The First Hospital of Jilin University, 130061 Changchun, China

**Keywords:** CNS cancer, Cancer therapeutic resistance, Chemotherapy

## Abstract

Temozolomide (TMZ) is the mainstream chemotherapeutic drug for treating glioblastoma multiforme (GBM), but the intrinsic or acquired chemoresistance to TMZ has become the leading clinical concern, which is related to the repair of DNA alkylation sites by O^6^-methylguanine-DNA methyltransferase (MGMT). Pyrvinium pamoate (PP), the FDA-approved anthelminthic drug, has been reported to inhibit the Wnt/β-catenin pathway within numerous cancer types, and Wnt/β-catenin signaling pathway can modulate the expression of MGMT gene. However, whether PP affects the expression of MGMT and enhances TMZ sensitivity in GBM cells remains unclear. In the present study, we found that PP and TMZ had synergistic effect on inhibiting the viability of GBM cells, and PP induced inhibition of MGMT and enhanced the TMZ chemosensitivity of GBM cells through down-regulating Wnt/β-catenin pathway. Moreover, the overexpression of MGMT or β-catenin weakened the synergy between PP and TMZ. The mechanism of PP in inhibiting the Wnt pathway was indicated that PP resulted in the degradation of β-catenin via the AKT/GSK3β/β-catenin signaling axis. Moreover, Ser552 phosphorylation in β-catenin, which promotes its nuclear accumulation and transcriptional activity, is blocked by PP that also inhibits the Wnt pathway to some extent. The intracranial GBM mouse model also demonstrated that the synergy between PP and TMZ could be achieved through down-regulating β-catenin and MGMT, which prolonged the survival time of tumor-bearing mice. Taken together, our data suggest that PP may serve as the prospect medicine to improve the chemotherapeutic effect on GBM, especially for chemoresistant to TMZ induced by MGMT overexpression.

## Introduction

Glioblastoma multiforme (GBM) is the most frequently occurring and aggressive primary intracranial malignancy with great mortality [1, 2]. Currently, temozolomide (TMZ) is a first-line clinical chemotherapeutic that has been widely used to treat GBM by causing methylation at various sites on DNA bases [3–5]; of them, the O^6^ methylation of guanine will elicit DNA damage, resulting in arrest of the cell cycle and eventually cell death [6, 7]. As a DNA repair enzyme, O^6^-methylguanine-DNA methyltransferase (MGMT) allows for the direct and rapid repair of those cytotoxic lesions resulted from TMZ, which is achieved through eliminating methyl from O^6^-methylguanine, finally reversing the DNA injury [3]. The high MGMT level in GBM contributes to the formation of chemoresistance, which is the leading cause of treatment failure [8, 9]. Therefore, MGMT is an important target for developing agents to recover the TMZ sensitivity [10, 11].

**Fig. 1:**
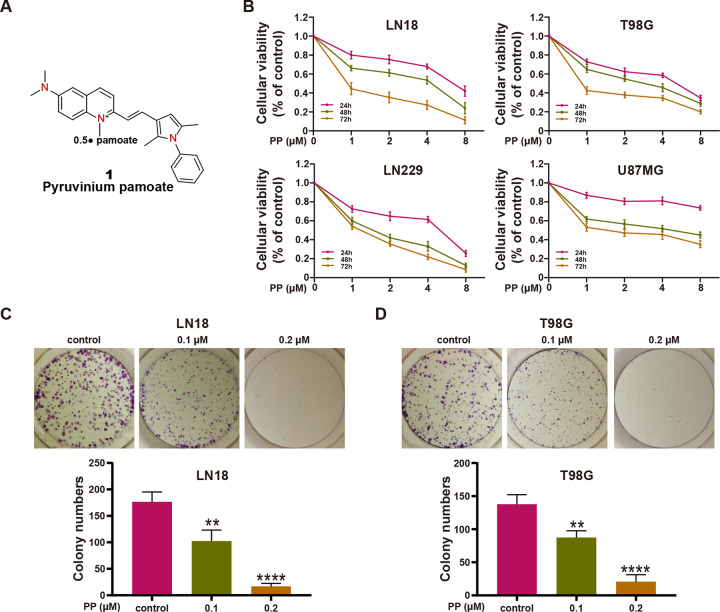
PP inhibited the viability and colony forming capacity in GBM cells. **A** PP chemical structure. **B** PP (0–8 μM) was used to treat the LN18, T98G, LN229, and U87MG GBM cell lines for 24, 48, and 72 h; then, MTT assay was conducted to detect cell viability (*n* = 3). **C**, **D** LN18 (**C**) and T98G (**D**) cells were subjected to PP treatment for 10 days. Later, the colonies formed were photographed and counted, as displayed in the histogram (*n* = 3). The results were presented in the manner of mean ± SD. Compared with control group, **p < 0.01; ****p < 0.001.

Pyrvinium pamoate (PP) (Fig. 1A) is a classical anthelmintic agent, which has been approved by the FDA in the 1950s to be used to treat pinworm infection [12]. Recent studies show that PP possesses various novel bioactivities, such as anti-adipogenic, antifungal, antibacterial, and antivirus activities [13–16]. Moreover, some studies have paid great attention to PP since it has strong anti-tumor effects on some cancer types, including urothelial carcinoma of bladder, hematological malignancies, breast cancer, colon cancer, and prostate cancer [12]. However, it remains unclear about whether PP enhances TMZ sensitivity in GBM cells.

β-catenin is the pivotal downstream effector in the canonical Wnt signaling pathway, which combines with the LEF/TCF transcription factors (TFs) complex in cell nucleus to activate the expression of target gene [17, 18]. Besides, it takes part in tumor genesis, development, immunity, dormancy, and maintenance of cancer stem cells [19, 20]. It is suggested that, β-catenin activity is mainly regulated at protein degradation level [21]. In addition, β-catenin is targeted for ubiquitination by a multi-protein destruction complex, which mainly includes Axin, CK, APC, and GSK-3β; as a result, it is degraded through proteasome-associated pathway [22]. PP can suppress the Wnt/β-catenin signaling pathway, but the detailed mechanism has not been thoroughly explored [23–25]. It has been confirmed the Wnt/β-catenin signaling pathway can modulate the expression of MGMT gene within the context of malignancies [26, 27]. Therefore, we speculated that PP might suppress the expression of MGMT to reduce the TMZ resistance by down-regulating the β-catenin signaling pathway within GBM. To verify this hypothesis, experiments were conducted in the current work to investigate whether PP reduced TMZ resistance and explore the possible mechanisms.

## Results

### PP inhibited the GBM cell viability and colony-forming capacity

To explore the PP role in GBM cell proliferation in vitro, MTT assay was performed to assess the viability rates of LN18, T98G, LN229, and U87MG cells. According to Figs. 1B and S1, significant reduction was observed in cell viability as the PP content and incubation time increased. Besides, the half maximal inhibitory concentration (IC50) values after 48 h of PP treatment were 3.04 μmol/L, 2.64 μmol/L, 1.48 μmol/L, and 4.09 μmol/L in LN18, T98G, LN229, and U87MG cell lines, respectively. Besides, the colony-forming capacities of LN18 and T98G cells in the presence or absence of PP were assessed for 10 days, which suggested markedly inhibited colony forming capacities in these two kinds of cells under PP treatment in a dose-dependent manner (Fig. 1C, D). The above findings indicated the effect of PP on suppressing the human GBM cells viability in vitro.

### PP promoted GBM cell apoptosis and resulted in the arrest of cell cycle

**Fig. 2:**
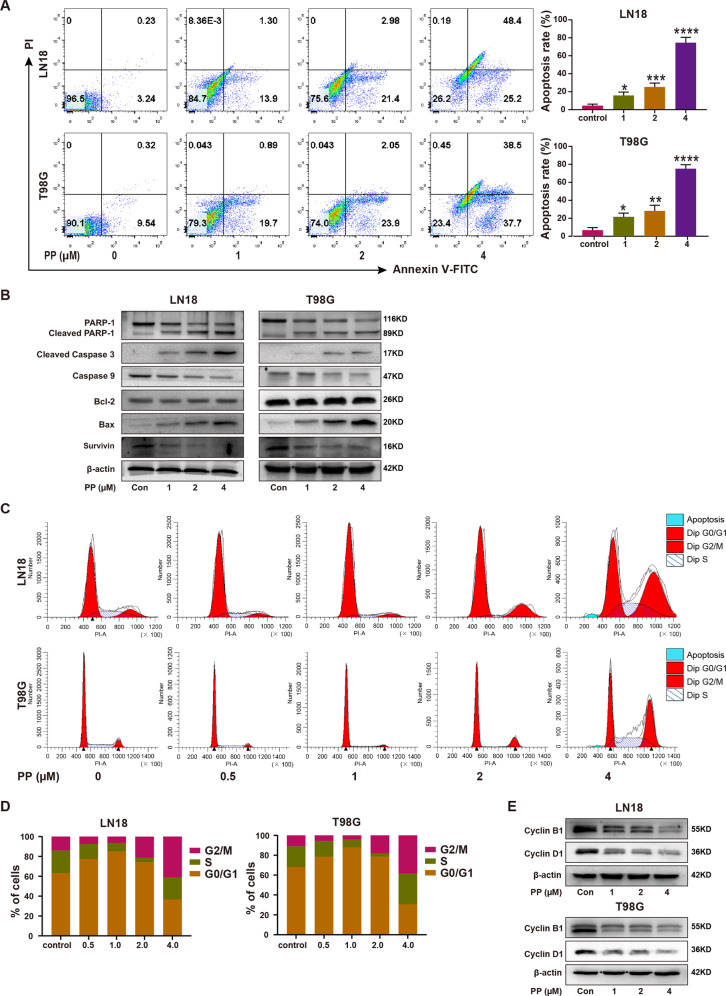
PP induced GBM cells apoptosis and the arrest of cell cycle. **A** LN18 and T98G cells were subjected to PP treatment at various concentrations for 48 h, and then flow cytometry was carried out along with annexin V-FITC and PI staining (left panel). The elevated apoptotic cells were quantified and presented in the histogram (right panel) (*n* = 3). **B** The protein levels of PARP-1, Bcl-2, Bax, caspase-9, cleaved caspase-3, and survivin were evaluated through western blotting following PP treatment at different contents. **C** PI staining combined with flow cytometry showed cell cycle distribution in the LN18 and T98G cell lines following PP treatment at different contents. **D** Histograms showed LN18 and T98G cell percentages at G0/G1, S as well as G2/M phase, respectively. **E** The cyclin B1 and cyclin D1 protein expression within LN18 and T98G cell lines following PP treatment for 48 h. The results were presented in the manner of mean ± SD. Compared with control group, *p < 0.05; **p < 0.01; ***p < 0.005; ****p < 0.001.

Cell apoptosis was examined through flow cytometry with annexin V/ PI analysis following 48 h of PP treatments. According to Fig. 2A, PP markedly promoted the apoptosis of GBM cell lines in dose-dependent way. Moreover, PP up-regulated apoptotic protein expression, including Bax, cleaved caspase-3, and cleaved PARP-1, but down-regulated survivin expression, an anti-apoptotic protein, and caspase 9. However, the expression of Bcl-2 did not change after PP treatment (Fig. 2B). These results suggested that PP promoted apoptosis in GBM cells through modulating the expression of proteins related to apoptosis.

For exploring whether PP affected the cell cycle, flow cytometry was carried out to analyze the DNA content of LN18 and T98G cell. Briefly, the LN18 and T98G cell lines were subjected to 48 h of PP treatments at different doses. As shown in Fig. 2C, D, PP induced the cell cycle arrest in a concentration-dependent way. Relative to the control group, PP processing at the doses of 0.5 μM and 1.0 μM slightly increased the cell proportion at G0/G1 phase, while PP treatment at 2.0 μM and 4.0 μM markedly increased the cell proportion at G2/M phase. Western blotting was performed to examine proteins related to cell cycle, which showed that PP down-regulated cyclin D1 and cyclin B1 expression as the concentration increased (Fig. 2E). Cyclin D1 has been extensively recognized as an essential regulator of the G1/S transition [28], whereas cyclin B1 regulates G2/M progression [29]. The above findings demonstrated that, PP reduced cyclin B1 and cyclin D1 expression within GBM cells, resulting in cell cycle arrest at G0/G1 and G2/M phases.

### PP down-regulated MGMT level to enhance the chemosensitivity of GBM cells to TMZ

**Fig. 3:**
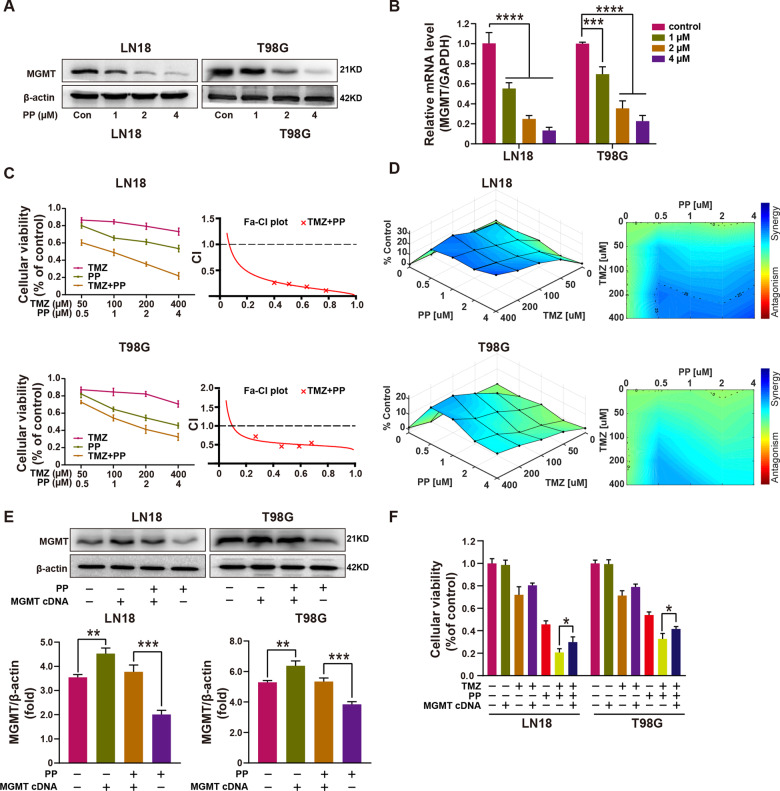
PP down-regulated MGMT expression and enhanced the TMZ chemotherapeutic effect on GBM cell lines. **A** The PP role in MGMT protein level was measured through western blotting. **B** The PP role in MGMT mRNA level was measured through quantitative RT-PCR (*n* = 3). **C** LN18 and T98G cell viability under PP and TMZ treatment at different contents (*n* = 3). CalcuSyn 2.0 was adopted to generate the curves. The Fa-CI plots showed the value of combination index (CI) in every fractional effect, and PP was synergized with TMZ (CI < 1). **D** Surface plots of LN18 and T98G cells treated with TMZ, PP, or combined TMZ + PP shown in 2D and 3D. Plots were generated using Combenefit program by applying HSA model. **E** MGMT protein levels in over-expressing MGMT and control cells were examined within LN18 and T98G cells (top panel). Densitometric analysis of MGMT/β-actin expression fold change (bottom panel) (*n* = 3). **F** Effect o**f** MGMT overexpression on cell viability under TMZ, PP, or TMZ combined PP treatment (*n* = 3). Results were presented in the manner of mean ± SD. *p < 0.05; **p < 0.01; ***p < 0.005; ****p < 0.001.

MGMT, one of the DNA repair proteins, removes the cytotoxic lesions caused by TMZ [30]; besides, it is a key factor for TMZ-resistance [31–33]. Therefore, we determined whether PP changed MGMT expression in GBM cells. Western blotting and quantitative real-time PCR analysis (qRT-PCR) showed that, the MGMT protein and mRNA expression was inhibited by PP within LN18 and T98G cell lines depending on its concentration (Fig. 3A, B). We further investigated whether PP enhanced the TMZ-mediated inhibitory effects on the viability of GBM cells. To this end, both LN18 and T98G cells, which displayed up-regulated MGMT expression and TMZ resistance, were subjected to 48 h of PP and TMZ treatment. As suggested by the results, PP combined with TMZ treatment suppressed cell viability in comparison with that of individual drugs alone (Fig. S2). Moreover, the CI values were determined to examine the synergy of combined treatment. As shown in Fig. 3C, PP showed synergy with TMZ (CI < 1) for both cell lines. Figure 3D represents the surface response of TMZ and PP on LN18 cells in a 5 × 5 checkerboard design. The colored areas denote the degree of synergism. As the highest single agent (HSA) model synergy and antagonism surface map shows, the interaction between TMZ and PP was almost likely additive/synergistic over the whole dose-response matrix, with the strongest synergistic effect centered at the concentration of 4 µM of PP and 400 µM of TMZ in LN18 cells, and 0.5 µM of PP and 200 or 400 µM of TMZ in T98G cells. The above findings indicated that, PP down-regulated MGMT expression but enhanced the chemosensitivity of GBM cells to TMZ.

For better exploring the MGMT effect on the PP-induced enhanced anti-tumor effect of TMZ chemotherapy, the scrambled control or MGMT cDNA plasmid was transfected into LN18 and T98G cells. MGMT protein expression increased with the MGMT overexpression within both cell lines either in the presence or absence of PP treatment (Fig. 3E). Furthermore, as suggested by MTT assay, MGMT overexpression restored reduced cell viability resulted from PP combined TMZ treatment (Fig. 3F). Taken together, the above findings suggested that, the PP-induced enhancement of TMZ chemotherapy depended on MGMT within GBM cells.

### PP inhibited the Wnt/β-catenin signaling pathway within the GBM cells

**Fig. 4:**
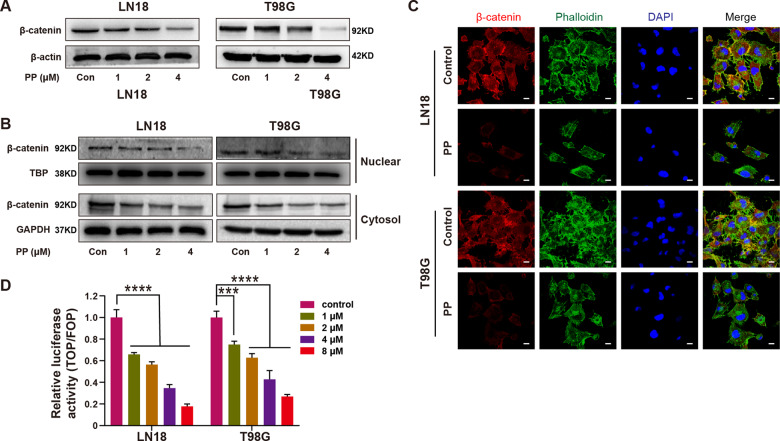
PP inhibited the Wnt/β-catenin signaling pathway within the GBM cells. **A** The PP role in β-catenin protein level was measured through western blotting. **B** Expression of β-catenin in the cytoplasm or nucleus was examined by western blotting following PP treatment at different contents. **C** β-catenin expression within LN18 and T98G cells following 2.0 μM PP treatment examined by immunofluorescence. The scale bar is 20 µm. **D** TCF/LEF-dependent luciferase activity was measured following PP treatment for 24 h (*n* = 3). The results were presented in the manner of mean ± SD. ***p < 0.005; ****p < 0.001.

PP is suggested to inhibit the Wnt/β-catenin signaling pathway within certain cancer types [12, 24, 34–36]. Therefore, the PP-caused alterations in the Wnt/β-catenin signaling pathway were explored within the GBM cell lines. In brief, the LN18 and T98G cells were exposed to PP for 48 h, and western blotting analysis showed that the total protein expression of β-catenin was down-regulated depending on PP concentration (Fig. 4A). Given that the β-catenin nuclear translocation is necessary to execute its roles in switching on target genes, the β-catenin levels in nucleus and cytoplasm were examined, respectively. According to Fig. 4B, C, the level of β-catenin either in cytoplasm or nucleus decreased within LN18 and T98G cells following 48 h of PP treatment. Consistently, the immunofluorescent results also verified that, β-catenin expression decreased within cytoplasm and nucleus following PP treatment (Fig. 4C). Besides, the changes in the Wnt/β-catenin-mediated transcription were also examined through the TOPflash/FOPflash dual-luciferase reporter assay on TCF/LEF binding sites. Compared with control, PP inhibited the TOPflash luciferase activity in LN18 and T98G cells depending on its content (Fig. 4D). Such findings indicated that, PP significantly inhibited the Wnt/β-catenin signaling pathway within GBM cell lines.

### β-catenin modulated the PP-caused suppression of MGMT and enhanced GBM cell chemosensitivity to TMZ

**Fig. 5:**
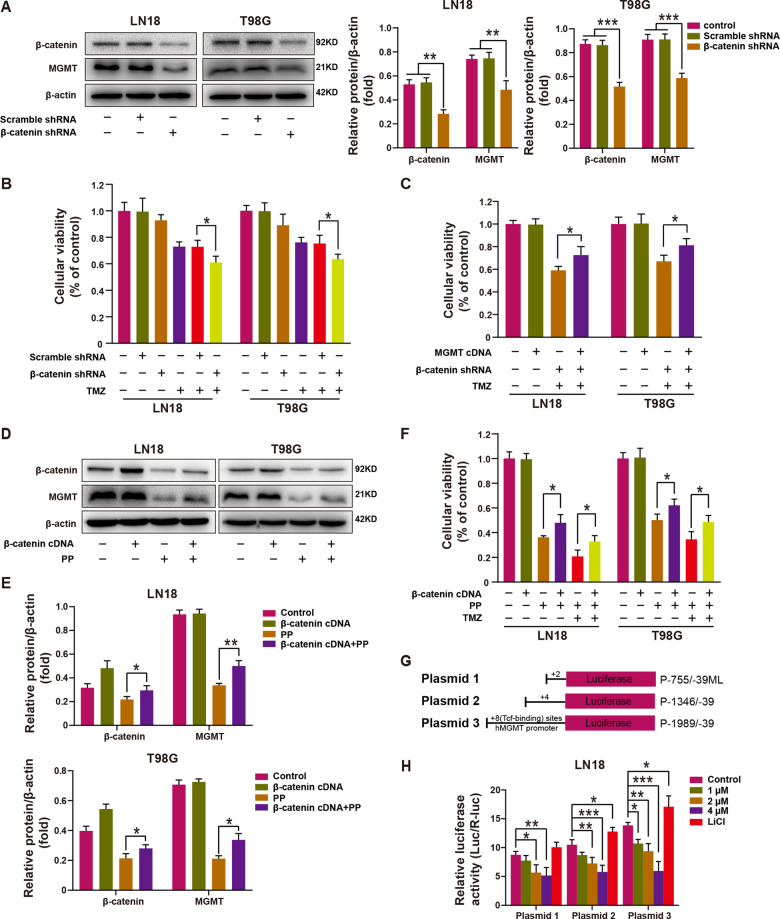
PP-induced MGMT suppression and weakened-resistance to TMZ of GBM cells mediated by β-catenin. **A** MGMT and β-catenin protein levels in β-catenin knockdown and control cells were examined within LN18 and T98G cells (left panel). Densitometric analysis of β-catenin and MGMT expression (right panel) (*n* = 3). **B** Cell viability in β-catenin knockdown and control cells under TMZ treatment within LN18 and T98G cells (*n* = 3). **C** Effect of MGMT overexpression on cell viability in β-catenin knockdown and TMZ treatment groups within LN18 and T98G cells (*n* = 3). **D** MGMT and β-catenin protein levels in β-catenin overexpression and control cells under PP treatment. **E** Densitometric analysis of β-catenin/β-actin and MGMT/β-actin expression relative fold change in LN18 (top panel) and T98G cells (bottom panel) (*n* = 3). **F** Effect of β-catenin overexpression on cell viability under PP or PP combined TMZ treatment within LN18 and T98G cells (*n* = 3). **G** Schematic diagram for the MGMT promoter luciferase reporter plasmids (P-755/−39 ML, P-1346/−39 ML, and P-1989/−39 ML) possessed desired candidate Tcf/Lef-binding site numbers. **H** MGMT promoter luciferase reporter activity was measured following PP treatment at different contents in LN18 cells (*n* = 3). Data were presented in the manner of mean ± SD. *p < 0.05; **p < 0.01; ***p < 0.005.

For exploring the role of β-catenin in regulating MGMT level as well as the GBM cell chemosensitivity to TMZ, both LN18 and T98G cells were transfected with scrambled or β-catenin shRNA. According to Figs. 5A and S3, knockdown of β-catenin significantly down-regulated the mRNA and protein expression of MGMT in both cell lines. Moreover, as suggested by the results of MTT assay, β-catenin knockdown boosted TMZ-induced decline in viability of cells (Fig. 5B). In addition, overexpression of MGMT partially restored the decline in cell viability caused by the synergistic effect of TMZ and β-catenin shRNA (Fig. 5C). Hence, according to the above data, β-catenin played a role in regulating MGMT level as well as GBM cell chemosensitivity to TMZ.

To examine the β-catenin effect on the PP-caused MGMT suppression and the enhancement in TMZ chemosensitivity, scrambled control or β-catenin cDNA was transfected into LN18 and T98G cells. β-catenin overexpression partially restored MGMT and β-catenin down-regulation induced by PP (Fig. 5D, E). In addition, β-catenin overexpression also partly restored the PP-caused down-regulated cellular viability and MGMT mRNA expression of LN18 and T98G cells (Figs. 5F and S4). Moreover, β-catenin overexpression offset the synergy of PP with TMZ within the two cell lines (Fig. 5F). For better understanding the underlying mechanisms of β-catenin in regulating the transcription of MGMT, and β-catenin effect on the PP-induced MGMT inhibition, the luciferase reporter gene plasmids, which were the three human MGMT promoter region plasmids containing different numbers of Tcf/Lef binding sites (Fig. 5G), were co-transfected with internal control plasmid pRL-TK into LN18 cells, respectively. As shown in Fig. 5H, the luciferase activity increased with the increase in Tcf/Lef-binding site numbers; besides, activating the Wnt signaling with LiCl enhanced the luciferase activity, whereas PP-induced β-catenin inhibition weakened the luciferase activity depending on the PP content. Taken together, it suggested that PP inhibited MGMT to enhance the TMZ chemosensitivity of GBM cells mediated by β-catenin.

### PP increased β-catenin degradation and suppressed the β-catenin phosphorylation on Ser-552

**Fig. 6:**
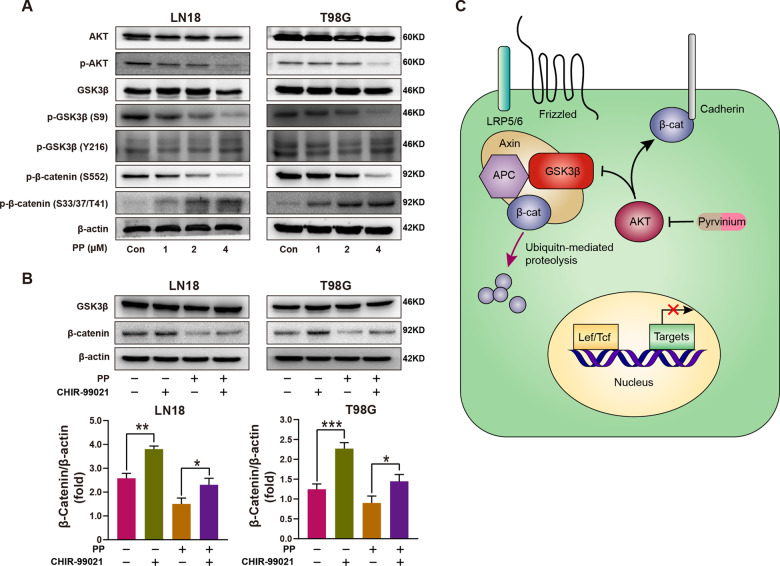
The underlying mechanism of PP in regulating β-catenin. **A** The protein levels of AKT, p-AKT, GSK3β, p-GSK3β (S9), p-GSK3β (Y216), p-β-catenin (S552), and p-β-catenin (S33/37/T41) were evaluated through western blotting following PP treatment at different contents. **B** Effect of pretreatment with GSK3β inhibitor CHIR-99021 on PP-induced β-catenin down-regulation (top panel). Densitometric analysis of β-catenin/β-actin expression fold change (bottom panel). **C** Schematic diagram for the underlying mechanism by which PP regulated β-catenin. PP inhibits the activation of AKT, thereby increasing the activity of GSK3β, leading to increased degradation of β-catenin by multi-protein destruction complex, which mainly includes Axin, APC, and GSK-3β. β-catenin has at least two distinct pools. One is located in cytoplasm which binds with E-cadherin, and the other one is involved in the Wnt signaling pathway so that shuttles between cytoplasm and nucleus [45]. AKT mediates the phosphorylation of β-catenin associated with plasma membrane, and then induces the dissociation of β-catenin from E-cadherin, which accumulates in cytoplasm and nucleus [30]. In the case of PP addition, PP reduces the p-AKT protein level and further down-regulates p-β-catenin (S552) expression in the cells. Consequently, dissociation of β-catenin from E-cadherin within cell membrane is reduced, so that the combination of β-catenin with the LEF/TCF transcription factors in cell nucleus is reduced, either, which hinders the activation of MGMT gene. *p < 0.05; **p < 0.01; ***p < 0.005.

β-catenin metabolism is mainly controlled by ubiquitination and proteasomal degradation [37–40]. Typically, GSK3β is an important β-catenin regulator in the upstream canonical Wnt/β-catenin signal transduction pathway [41]; moreover, the phosphorylation modification of GSK3β on the S33/37/T41 sites in β-catenin is a prerequisite for ubiquitination degradation [42, 43]. Therefore, the impacts of PP on phospho-β-catenin (p-β-catenin, S33/37/T41) and GSK3β were investigated. According to Fig. 6A, p-β-catenin (S33/37/T41) increased in PP-treated GBM cells depending on PP content, and such results reflected the enhanced activity of GSK3β for β-catenin. Moreover, results of western blotting revealed that, the total GSK3β and GSK3β at the up-regulatory site Y216 remained largely unchanged, but the phosphorylation of GSK3β at its down-regulatory site S9 decreased. Since GSK-3β was phosphorylated by AKT at Ser9, the effect of PP on AKT was subsequently analyzed. As shown in Fig. 6A, p-AKT was significantly down-regulated in the PP-treated GBM cells, which was consistent with the result of GSK3β (S9) down-regulation. Moreover, after 24 h of pretreatment with 2 μmol/L CHIR-99021 (the GSK-3β inhibitor), the LN18 and T98G cells were exposed to PP for 48 h. According to Fig. 6B, the decrease in β-catenin caused by PP was partially recovered by CHIR-99021 pretreatment. Therefore, these results indicated that PP suppressed the Wnt/β-catenin signaling pathway through degrading β-catenin.

Previous studies show that Ser552 of β-catenin can be phosphorylated by AKT, and this results in β-catenin disassociation from the contacts between cells, increasing the transcriptional activity of β-catenin and promoting tumor cell invasion [44]. Therefore, we investigated whether PP regulated the phosphorylation of β-catenin at Ser552. According to Fig. 6A, p-β-catenin (S552) was down-regulated within the PP-treated GBM cells depending on PP concentration. Taken together, we demonstrated a model regarding the PP effect on the non-canonical and canonical Wnt/β-catenin signaling pathway (Fig. 6C).

### PP suppressed GBM growth and was synergized with TMZ in vivo

**Fig. 7:**
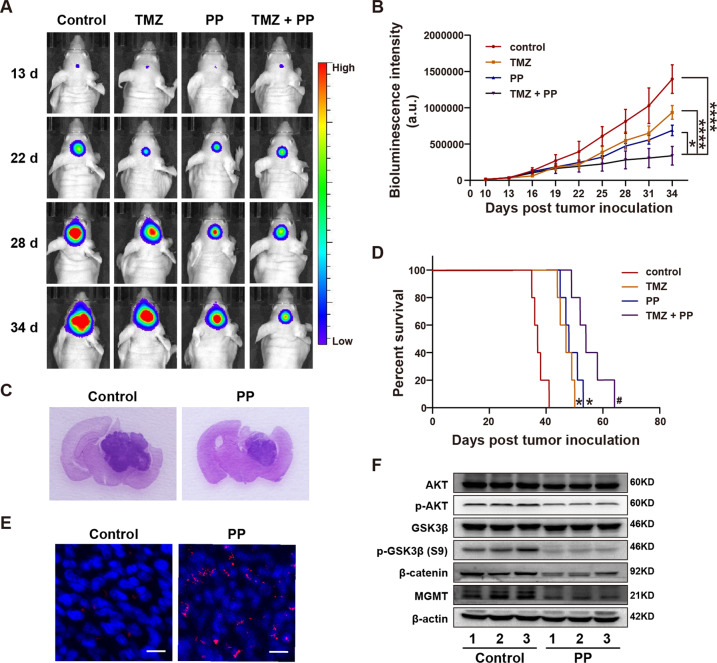
PP suppressed GBM xenograft growth and was synergized with TMZ in vivo. **A** Typical mouse bioluminescence images at 13, 22, 28 and 34 days following GBM cell implantation. **B** The bioluminescence images were quantitatively analyzed for four experimental groups (*n* = 5). *p < 0.05; ****p < 0.001. **C** Representative images of HE-stained brain sections in PP and control group. **D** Kaplan−Meier survival curves for the four experimental groups (*n* = 5). **E** Representative imag**e**s of TUNEL staining in PP and control group. The scale bar is 10 µm. **F** The protein levels o**f** p-AKT, p-GSK3β (S9), MGMT, and β-catenin were evaluated through western blotting within orthotopic GBM xenograft (*n* = 3). The results were displayed in the manner of mean ± SD. *p < 0.05 vs. control group; ^#^*p* < 0.05 vs. single group.

The intracranial orthotopic GBM xenograft mouse model was established for evaluating the effect and tolerability of PP in vivo. U87MG-Luc cells were injected into brain hippocampus. One week later, the mice were randomized as four groups, including control (vehicle), intraperitoneal injection of PP at 0.5 mg/kg/day for 3 consecutive weeks, gavage of TMZ at 20 mg/kg/day for 3 consecutive weeks, and PP at 0.5 mg/kg combined with 20 mg/kg TMZ for 3 consecutive weeks. Bioluminescence imaging (BLI) and hematoxylin-eosin (HE) staining of brain tumor sections revealed the markedly reduced tumor volume in PP treatment group compared with control group (Fig. 7A–C). In addition, PP treatment dramatically extended the survival of tumor-bearing mice (Fig. 7D). Furthermore, TUNEL staining showed that, PP treatment group had remarkably increased cell apoptosis compared with control group (Fig. 7E). Additionally, as suggested by western blotting, PP treatment down-regulated the protein levels of p-GSK-3β (S9), p-AKT, MGMT, and β-catenin of the tumor tissue, which conformed to results obtained in vitro (Fig. 7F). These observations suggested that PP had anti-GBM effect in vivo. Furthermore, the synergistic anti-tumor effect of PP with TMZ in vivo was also explored. According to Fig. 7B, D, PP combined with TMZ treatment notably extended the median survival for tumor-bearing mice and inhibited tumor growth compared with those in single drug treatment groups. To sum up, the above results indicated that, PP suppressed GBM growth and was synergized with TMZ in vivo.

## Discussion

At present, TMZ is verified as the only chemotherapeutic that remarkably extend the overall survival (OS) for GBM [5, 11]. However, the resistance to TMZ therapy is a leading cause responsible for the failed treatment; on this account, reversing the chemoresistance of TMZ is the key to enhance the therapeutic results for GBM [8, 11]. It is well-known that, it is a time-consuming and costly process to discover and develop a new anti-cancer agent [45]. Therefore, the repurposing of drugs, which indicates to use the available drugs for a novel disease indication, is promising to have fast clinical effect at the low costs relative to the development of a new drug [46]. PP, which is a classical anthelmintic agent, has been reported to cause strong anti-tumor effect in the GBM stem-like cells (GSC) of GBM. PP could attenuate self-renewal and proliferative capacities of glioblastoma-initiating cells (GICs) in part through inhibition of Wnt/β-catenin signaling and other essential stem cell regulatory pathways [47]. In addition, PP could induce GSC-selective cytotoxicity by inhibiting mitochondrial function [48] or promoting methyl CpG binding domain protein 3 (MBD3) protein degradation, which plays a critical role in stem cell pluripotency, differentiation, and cell death [49].

This study first examined the individual drug PP effect on GBM. The results suggested that, PP obviously inhibited GBM cell proliferation but induced apoptosis through up-regulating pro-apoptotic proteins cleaved caspase-3, cleaved PARP-1 and Bax, while down-regulating the anti-apoptotic protein survivin. Besides, expression of Bcl-2, the anti-apoptotic protein, was not affected. On the other hand, the Bax/Bcl-2 ratio may determine the susceptibility of cells to apoptosis following an apoptotic stimulus [50]. Therefore, we speculated that, an elevated Bax/Bcl-2 ratio determined the PP-induced GBM cell apoptosis. PP resulted in the arrest of cell cycle through decreasing the expression of cyclin B1 and cyclin D1. Moreover, PP obviously decreased the tumor volume and extended the median survival of tumor-bearing nude mice. GBM cells and xenograft GBM models were used to further examine the synergistic effect of PP and TMZ. Our study was the first to suggest that, PP enhanced the chemosensitivity of GBM to TMZ both in vitro and in vivo through down-regulating MGMT gene expression. The observations indicated that, PP reduced the GBM chemoresistance to TMZ in a MGMT-dependent manner.

Aberrant activation of the Wnt/β-catenin signaling pathway frequently occurs among various type of cancers, including GBM [51–53]. The excessively activated Wnt signaling makes key contributions to GBM cell proliferation, metastasis, and invasion, as well as angiogenesis and chemoresistance [54–56]. Therefore, Wnt/β-catenin signaling pathway is the fascinating putative target for novel anti-GBM agents and enhancing the sensitivity to TMZ. According to our results in this study, knockdown of β-catenin induced MGMT down-regulation in the meantime of enhancing the chemosensitivity of GBM cell lines to TMZ. Such result is consistent with previous studies that the Wnt/β-catenin signaling pathway modulate the expression of MGMT gene, while suppressing the Wnt signaling enhances the TMZ sensitivity in GBM [26, 27]. We also found that PP significantly decreased the β-catenin expression in cytoplasm and nucleus. β-catenin overexpression partly reversed the PP suppression effect on GBM cell viability and down-regulation of MGMT, thus increasing TMZ resistance in GBM. The above observations indicated that, the PP-induced MGMT suppression and TMZ sensitization were dependent on β-catenin. Also, it was discovered that, PP reduced the activity of MGMT promoter reporter gene, further suggesting the effect of β-catenin on activating MGMT expression via directly interacting with the binding sites of Tcf/Lef transcriptional factor at the MGMT gene promoter region.

Accumulating evidences suggests that, PP inhibits the Wnt signaling pathway and decreases β-catenin level in some cancer types [12, 34–36, 57], but the mechanism remains unclear so far. Our data showed that, PP up-regulated p-S33/37/T41 β-catenin expression, which is necessary to degrade β-catenin under the control of ubiquitin [23, 58]. Reduction in p-GSK3β at S9 leads to increased GSK3β activity, which may increase the expression of p-S33/37/T41 β-catenin [23, 59]. Besides, the Ser9 site of GSK3β is the phosphorylation site for AKT [60]. This study further suggested the protein level of p-AKT dramatically declined in the PP-treated GBM cells. Based on the above results, we inferred that, PP suppressed the Wnt signaling via the AKT/GSK3β/β-catenin signaling axis.

β-catenin is a dual function protein that has at least two distinct pools, among which, one binds with E-cadherin and is located in cytoplasm, while the other one participates in the Wnt signaling and shuttles between cytoplasm and nucleus [20, 61]. AKT-mediated phosphorylation of β-catenin at the Ser552 site of the pool related to plasma membrane induces the disassociation of β-catenin from E-cadherin, together with cytoplasmic and nuclear accumulation [44, 62]. This may be another way that Akt crosstalks with the Wnt signaling pathway. Our results suggested that, PP reduced the p-AKT protein level and further down-regulated p-β-catenin (S552) expression within GBM cells. Consequently, we hypothesized that, PP induced the reduced β-catenin dissociation from E-cadherin within cell membrane, which partially inhibited the Wnt pathway.

To sum up, this work has first demonstrated that, PP enhances the GBM chemosensitivity to TMZ both in vivo and in vitro. Further, PP mediates MGMT suppression through β-catenin to achieve TMZ sensitization. Additionally, it should be noted that, the effect of PP on suppressing the Wnt signaling pathway may be mediated through β-catenin degradation and anchoring with cell membranes. The above observations suggest the potential of using PP as a TMZ chemotherapy sensitizer in GBM treatment.

## Materials and methods

### Reagents

PP was provided by MedChem Express (Princeton, NJ, USA). CHIR-99021 was provided by Selleckchem Company (Houston, TX, USA). TMZ and lithium chloride were provided by Sigma (St. Louis, MO, USA). The anti-Cleaved Caspase-3, anti-Caspase 9, anti-Bcl-2, anti-Bax, anti-Survivin, anti-cyclin B1, anti-cyclin D1, anti-β-catenin, anti-p-β-catenin (S552), anti-p-β-catenin (S33/37/T41), anti-GSK-3β, anti-p-GSK-3β (Ser9), anti-AKT, anti-p-AKT, anti-TBP, anti-GAPDH, and anti-histone H3 antibodies were provided by Cell Signaling Technology (Beverly, MA, USA). The anti-MGMT and anti-β-actin antibodies were provided by Abcam (Cambridge, MA, USA). The anti-p-GSK3β (Y216) antibody was provided by Thermo (Invitrogen, CA, USA). The anti-PARP-1 antibody was provided by Santa Cruz Biotechnology (Santa Cruz, CA, USA).

### Cell viability and cell colony formation assay

The human LN18, T98G, LN229, and U87MG GBM cells, provided by ATCC, were cultured in DMEM containing 100 μg/mL streptomycin, 100 U/mL penicillin, and 10% fetal bovine serum (FBS) under 5% CO2 and 37 °C conditions. STR profiling was used to authenticate GBM cells and all the cells were verified none mycoplasma contamination. In all, 5 × 10^3^ cells/well were planted into 96-well plates to culture for a period of 12 h. Then, the selected medicine at specific contents was used to treat cells for specific times. Later, the MTT assay was carried out to measure cell viability, which was presented in the form of absorbance (%) in untreated cells at the wavelength of 570 nm.

The LN18 and T98G cells (1000 cells/well) were inoculated into six-well plates for 24 h and then treated with PP at indicated contents for 10 days. Later, these cells were subjected to 4% formaldehyde fixation and 0.2% crystal violet staining. Each well in the six-well plates was photographed and colonies that contained ≥50 cells were counted microscopically under ×4 magnification.

### Analysis of cell apoptosis and cell cycle

LN18 and T98G cells were treated with the indicated concentration of PP for 48 h and then collected. For cell apoptosis analysis, cells were stained with Annexin V and PI according to the manufacturer’s instructions of the Annexin V/PI Apoptosis Detection kit (Becton Dickinson, San Diego, CA, USA). Flow cytometry was performed and the data was analyzed using FlowJo Version 10.0 software. For the cell cycle analysis, those collected cells were subjected to 70% ethanol treatment under the temperature of −20 °C overnight, followed by 25 min of PI (BD) and RNase (BD) treatment in dark. The flow cytometry was conducted and ModFit LT 3.3 was adopted for data analysis.

### Western blotting analysis

The RIPA buffer (Beyotime Biotechnology, China) containing the phosphatase and protease inhibitors was employed to lyse the frozen GBM xenograft tissues and cultured GBM cells. Then BCA protein detection kit (Pierce, Rockford, IL, USA) was adopted to determine protein content. After 10 min of heating in the 100 °C water, the protein samples were isolated through SDS-PAGE, followed by transfer to the PVDF films (Millipore, Billerica, MA, USA). Afterwards, the films were blocked using 5% skim milk for 30 min under ambient temperature, incubated using appropriate primary antibodies under the temperature of 4 °C overnight, and then incubated using the secondary peroxidase-conjugated goat anti-rabbit antibody for 2 h under ambient temperature. Finally, the Odyssey Infrared Imaging System (LI-COR) was used to analyze the immunoblots.

### Quantitative real-time PCR analysis

The TRIzol (Invitrogen, Carlsbad, CA, USA) was utilized to isolate the total RNA based on GBM cells in accordance with manufacturer protocols. Later, the TransScript One-Step gDNA Removal and cDNA Synthesis SuperMix Kit (Transgen Biotech, Beijing, China) was used to prepare cDNA from the extracted RNA. Later, the SYBR green PCR master mix (TransGen Biotech, China) was utilized to perform qRT-PCR onto the MxPro-Mx3005 P Real-time PCR system (Agilent Technologies, USA). All primers used in this assay were prepared via Sangon Biotech (Shanghai, China), including:

GAPDH: 5′-ATCATCCCTGCCTCTACTGG-3′ (forward),

GAPDH: 5′-GTCAGGTCCACCACTGACAC-3′(reverse);

MGMT: 5′- GTTTTCCAGCAAGAGTCGTTC -3′ (forward),

MGMT: 5′- GCTGCTAATTGCTGGTAAGAAA-3′ (reverse).

GAPDH served as the internal reference.

### Synergy of drug combination

The type of interaction produced by TMZ and PP combination was performed using two methods, the isobologram analysis using CalcuSyn software version 2 (Biosoft, Cambridge, UK) and the dose-matrix approach using Combenefit software (Cancer Research, Cambridge, UK) according to the instructions [63, 64]. Combined interaction between PP and TMZ was determined according to the combination index (CI) for assessing the antagonistic effect (CI > 1), the additive effect (CI = 1) or the synergistic effect (CI < 1).

### Cell transfections

Lipofectamine 2000 (Invitrogen, Carlsbad, CA, USA) was used to transfect scrambled shRNA, β-catenin shRNA, scrambled cDNA, β-catenin cDNA, or MGMT cDNA (GeneChem, Shanghai, China) into the LN18 and T98G cell lines following manufacturer protocols. Thereafter, the original medium was discarded, while complete DMEM medium was added after 6 h of transfection. Later, cells were incubated with selected drug after transfection for 24 h, and they were then used in later analysis.

### Immunofluorescence staining

LN18 cells were subjected to 24 h of cultivation onto the uncoated glass slides, followed by another 24 h of PP treatment. The cells were washed with PBS, fixed with 4% paraformaldehyde for 20 min at 37 °C, permeabilized with 0.1% Triton X-100 for 10 min, and blocked in 5% BSA (Sigma) for 1 h at room temperature. The cells were immunostained with an antibody to β catenin (1:200, Cell Signaling Technology) at 4 °C overnight and subsequently incubated with FITC conjugated secondary antibodies (Pierce Biotechnology, Rockford, USA) for 0.5 h at room temperature in darkness. Cell nuclei was stained with DAPI (Invitrogen, Waltham, MA, USA), and the LSM 880 confocal laser scanning microscope (CLSM, Zeiss, Jena, Germany) was performed to visualize cells.

### TOPflash/FOPflash luciferase reporter assay

LN18 and T98G cells cultured in 24-well plates were co-transfected with 20 ng pRL-TK (Renilla-TK-luciferase vector; Promega, Madison, USA) and 500 ng of M51 Super 8 × FOPflash or M50 Super 8 × TOPflash (TOPflash mutant) plasmids for 24 h before another 24 h of PP treatment. Subsequently, dual-luciferase reporter assay was carried out in accordance with manufacturer protocols by the use of the assay kit (Promega).

### MGMT promoter reporter vector constructs and luciferase reporter assay

MGMT gene sequence was searched using NCBI Genome Database. The 717 bp, 1308 bp and 1951 bp fragments of MGMT promoter region were cloned to the pEASY-T5 Zero cloning vector (Transgen Biotech, Beijing, China), then cut with HindIII/XholI and subcloned at the HindIII/XholI site of PGL 4.1 basic vector to generate p-755/−39 ML, p-1346/−39 ML and p-1989/−39 ML, respectively. These above-mentioned 3 MGMT promoter constructs possessed 2, 4 and 8 candidate binding sites of TCF/LEF (Fig. 5I), respectively, as predicted by the JASPAR Predicted (http://jaspar.genereg.net/).

LN18 and T98G cell cultured in 24-wells plate were co-transfected with 500 ng/well MGMT promoter reporter plasmids and 20 ng/well pRL-TK (Promega, Madison, USA), followed by 24 h of PP or LiCl treatment. After 48 h of transfection, cells were harvested, and the detection kit (Promega) was used to measure the luciferase activity of the cells.

### Xenograft tumor model

The 8-week-old female BALB/c nude mice, provided by Charles River (Beijing, China), were kept under the specific pathogen-free environment with free access to food and water. The animal procedure conformed to the stipulations outlined in Guide for the Care and Use of Laboratory Animals. The Jilin University Animal Care and Use Committee had approved our study protocol. For establishing an intracranial brain tumor-bearing mouse model, U87MG-Luc cells (2 × 10^5^) were suspended in 2 μl PBS and injected into brain hippocampus. Tumor formation was verified by bioluminescence imaging (BLI) of all transplanted mice.

### Treatment of tumor-bearing mice

One week after tumor cells inoculation, the mice were randomized as four groups, including control (vehicle), intraperitoneal injection of PP at 0.5 mg/kg/day for 3 consecutive weeks, gavage of TMZ at 20 mg/kg/day for 3 consecutive weeks, and 0.5 mg/kg PP combined with 20 mg/kg TMZ (*n* = 5 mice per group). BLI was carried out to detect the intracranial tumor growth with IVIS Spectrum Live Imaging System (PerkinElmer, Branford, USA). The survival rate of mice in every group was observed.

In order to explore the mechanisms of PP on GBM in vivo, U87MG-Luc tumor-bearing mice were divided into two groups and intraperitoneally injected of PP at 0.5 mg/kg/day, or PBS for 3 consecutive weeks (*n* = 3 mice per group). All mice from the two groups were sacrificed 48 h after the last treatment (at day 30 after tumor implantation), and the brains or tumors of the mice in both groups were harvested for histopathology, TUNEL staining and Western blot analysis.

### Histopathology

Brains were collected from U87MG-luc tumor-bearing mice in the control and PP group after 3 consecutive weeks of treatment, then fixed in 4% paraformaldehyde and sliced into 10-μm-thick sections. Brain sections were stained by hematoxylin-eosin (H&E) and photographed.

### TUNEL staining

The tumor slides were subjected to xylene deparaffinage and gradient alcohol hydration, 4% paraformaldehyde fixation, and Proteinase K solution permeabilization in succession. Then, the in situ cell apoptosis detection kit (Invitrogen, Carlsbad, CA, USA) was utilized according to the manufacturer’s instructions to detect the stained slides using CLSM.

### Statistical analysis

All data obtained from at least three independent experiments and were expressed as mean ± standard deviation (SD). The differences between two groups were conducted using two-tailed unpaired Student’s *t* test and differences between more than two groups were compared by one-way ANOVA with Tukey’s multiple comparison. For analyzing the Kaplan–Meier survival curves, the Mantel–Cox test was used. Statistical analyses were carried out with GraphPad Prism 8.0 and *P*-values of <0.05 indicated that difference was statistically significant.

## Supplementary information

Figure S1

Figure S2

Figure S3

Figure S4

Supplementary information

## Data Availability

The datasets used and/or analyzed during the current study are available from the corresponding author on reasonable request.

## References

[1] Wen PY, Kesari S (2008). Malignant gliomas in adults. New Engl J Med.

[2] Omuro A, DeAngelis LM (2013). Glioblastoma and other malignant gliomas: a clinical review. JAMA.

[3] Jiapaer S, Furuta T, Tanaka S, Kitabayashi T, Nakada M (2018). Potential strategies overcoming the temozolomide resistance for glioblastoma. Neurol Med -Chir.

[4] Fulda S (2018). Cell death-based treatment of glioblastoma. Cell Death Dis.

[5] Lan F, Yang Y, Han J, Wu Q, Yu H, Yue X (2016). Sulforaphane reverses chemo-resistance to temozolomide in glioblastoma cells by NF-kappaB-dependent pathway downregulating MGMT expression. Int J Oncol.

[6] Fukai J, Koizumi F, Nakao N (2014). Enhanced anti-tumor effect of zoledronic acid combined with temozolomide against human malignant glioma cell expressing O6-methylguanine DNA methyltransferase. PLoS ONE.

[7] Hombach-Klonisch S, Mehrpour M, Shojaei S, Harlos C, Pitz M, Hamai A (2018). Glioblastoma and chemoresistance to alkylating agents: Involvement of apoptosis, autophagy, and unfolded protein response. Pharmacol Ther.

[8] Perazzoli G, Prados J, Ortiz R, Caba O, Cabeza L, Berdasco M (2015). Temozolomide resistance in glioblastoma cell lines: implication of MGMT, MMR, P-glycoprotein and CD133 expression. PLoS ONE.

[9] Karachi A, Dastmalchi F, Mitchell DA, Rahman M (2018). Temozolomide for immunomodulation in the treatment of glioblastoma. Neuro Oncol.

[10] Johnsen JI, Wickstrom M, Baryawno N (2016). Wingless/beta-catenin signaling as a modulator of chemoresistance in cancer. Mol Cell Oncol.

[11] Yi GZ, Huang G, Guo M, Zhang X, Wang H, Deng S (2019). Acquired temozolomide resistance in MGMT-deficient glioblastoma cells is associated with regulation of DNA repair by DHC2. Brain.

[12] Momtazi-Borojeni AA, Abdollahi E, Ghasemi F, Caraglia M, Sahebkar A (2018). The novel role of pyrvinium in cancer therapy. J Cell Physiol.

[13] Wang Z, Dai Z, Luo Z, Zuo C (2019). Identification of pyrvinium, an anthelmintic drug, as a novel anti-adipogenic compound based on the gene expression microarray and connectivity map. Molecules.

[14] Simm C, May RC (2019). Zinc and iron homeostasis: target-based drug screening as new route for antifungal drug development. Front Cell Infect Microbiol.

[15] Li T, Feng J, Xiao S, Shi W, Sullivan D, Zhang Y (2019). Identification of FDA-approved drugs with activity against stationary phase bartonella henselae. Antibiotics.

[16] Shen L, Niu J, Wang C, Huang B, Wang W, Zhu N (2019). High-throughput screening and identification of potent broad-spectrum inhibitors of coronaviruses. J Virol.

[17] Anastas JN, Moon RT (2013). WNT signalling pathways as therapeutic targets in cancer. Nat Rev Cancer.

[18] Clevers H, Nusse R (2012). Wnt/β-catenin signaling and disease. Cell.

[19] Shang S, Hua F, Hu ZW (2017). The regulation of beta-catenin activity and function in cancer: therapeutic opportunities. Oncotarget.

[20] Cui C, Zhou X, Zhang W, Qu Y, Ke X (2018). Is β-catenin a druggable target for cancer therapy?. Trends Biochem Sci.

[21] Krieghoff E, Behrens J, Mayr B (2006). Nucleo-cytoplasmic distribution of beta-catenin is regulated by retention. J Cell Sci.

[22] Xing Y, Clements WK, Kimelman D, Xu W (2003). Crystal structure of a beta-catenin/axin complex suggests a mechanism for the beta-catenin destruction complex. Genes Dev.

[23] Zheng L, Liu Y, Pan J (2017). Inhibitory effect of pyrvinium pamoate on uveal melanoma cells involves blocking of Wnt/beta-catenin pathway. Acta Biochim Biophys Sin.

[24] Zhang C, Zhang Z, Zhang S, Wang W, Hu P (2017). Targeting of Wnt/beta-catenin by anthelmintic drug pyrvinium enhances sensitivity of ovarian cancer cells to chemotherapy. Med Sci Monit.

[25] Cui L, Zhao J, Liu J (2018). Pyrvinium sensitizes clear cell renal cell carcinoma response to chemotherapy via casein kinase 1α-dependent inhibition of Wnt/β-catenin. Am J Med Sci.

[26] Bi Y, Li H, Yi D, Bai Y, Zhong S, Liu Q (2018). β-catenin contributes to cordycepin-induced MGMT inhibition and reduction of temozolomide resistance in glioma cells by increasing intracellular reactive oxygen species. Cancer Lett.

[27] Wickström M, Dyberg C, Milosevic J, Einvik C, Calero R, Sveinbjörnsson B (2015). Wnt/β-catenin pathway regulates MGMT gene expression in cancer and inhibition of Wnt signalling prevents chemoresistance. Nat Commun.

[28] Donjerkovic D, Scott DW (2000). Regulation of the G1 phase of the mammalian cell cycle. Cell Res.

[29] Huang Y, Sramkoski RM, Jacobberger JW (2013). The kinetics of G2 and M transitions regulated by B cyclins. PLoS ONE.

[30] Nikolova T, Roos WP, Krämer OH, Strik HM, Kaina B (2017). Chloroethylating nitrosoureas in cancer therapy: DNA damage, repair and cell death signaling. Biochim Biophys Acta-Rev Cancer.

[31] Wick W, Weller M, van den Bent M, Sanson M, Weiler M, von Deimling A (2014). MGMT testing−the challenges for biomarker-based glioma treatment. Nat Rev Neurol.

[32] Chen TC, Chan N, Minea RO, Hartman H, Hofman FM, Schönthal AH (2018). Rare stochastic expression of O6-methylguanine- DNA methyltransferase (MGMT) in MGMT-negative melanoma cells determines immediate emergence of drug-resistant populations upon treatment with temozolomide in vitro and in vivo. Cancers.

[33] Jackson CB, Noorbakhsh SI, Sundaram RK, Kalathil AN, Ganesa S, Jia L (2019). Temozolomide sensitizes MGMT-deficient tumor cells to ATR inhibitors. Cancer Res.

[34] Zheng L, Liu Y, Pan J (2017). Inhibitory effect of pyrvinium pamoate on uveal melanoma cells involves blocking of Wnt/β-catenin pathway. Acta Biochim Biophys Sin.

[35] Xu F, Zhu Y, Lu Y, Yu Z, Zhong J, Li Y (2018). Anthelmintic pyrvinium pamoate blocks Wnt/β-catenin and induces apoptosis in multiple myeloma cells. Oncol Lett.

[36] Barbarino M, Cesari D, Intruglio R, Indovina P, Namagerdi A, Bertolino FM (2018). Possible repurposing of pyrvinium pamoate for the treatment of mesothelioma: a pre-clinical assessment. J Cell Physiol.

[37] Li Z, Zhou L, Jiang T, Fan L, Liu X, Qiu X (2019). Proteasomal deubiquitinase UCH37 inhibits degradation of β-catenin and promotes cell proliferation and motility. Acta Biochim Biophys Sin..

[38] Kim B, Song TY, Jung KY, Kim SG, Cho EJ (2017). Direct interaction of menin leads to ubiquitin-proteasomal degradation of β-catenin. Biochem Biophys Res Commun.

[39] Chen C, Zhu D, Zhang H, Han C, Xue G, Zhu T (2018). YAP-dependent ubiquitination and degradation of β-catenin mediates inhibition of Wnt signalling induced by Physalin F in colorectal cancer. Cell Death Dis.

[40] Khan M, Muzumdar D, Shiras A (2019). Attenuation of tumor suppressive function of FBXO16 ubiquitin ligase activates Wnt signaling in glioblastoma. Neoplasia.

[41] Liang WC, Wong CW, Liang PP, Shi M, Cao Y, Rao ST (2019). Translation of the circular RNA circβ-catenin promotes liver cancer cell growth through activation of the Wnt pathway. Genome Biol.

[42] van Noort M, van de Wetering M, Clevers H (2002). Identification of two novel regulated serines in the N terminus of beta-catenin. Exp Cell Res.

[43] Alomar SY, Mansour L, Abuderman A, Alkhuriji A, Arafah M, Alwasel S (2016). β-Catenin accumulation and S33F mutation of CTNNB1 gene in colorectal cancer in Saudi Arabia. Pol J Pathol.

[44] Fang D, Hawke D, Zheng Y, Xia Y, Meisenhelder J, Nika H (2007). Phosphorylation of beta-catenin by AKT promotes beta-catenin transcriptional activity. J Biol Chem.

[45] Pushpakom S, Iorio F, Eyers PA, Escott KJ, Hopper S, Wells A (2019). Drug repurposing: progress, challenges and recommendations. Nat Rev Drug Discov.

[46] Basso J, Miranda A, Sousa J, Pais A, Vitorino C (2018). Repurposing drugs for glioblastoma: From bench to bedside. Cancer Lett.

[47] Venugopal C, Hallett R, Vora P, Manoranjan B, Mahendram S, Qazi MA (2015). Pyrvinium targets CD133 in human glioblastoma brain tumor-initiating cells. Clin Cancer Res.

[48] Datta S, Sears T, Cortopassi G, Woolard K, Angelastro JM, Repurposing FDA (2021). approved drugs inhibiting mitochondrial function for targeting glioma-stem like cells. Biomed Pharmacother.

[49] Moon BS, Cai M, Lee G, Zhao T, Song X, Giannotta SL (2020). Epigenetic modulator inhibition overcomes temozolomide chemoresistance and antagonizes tumor recurrence of glioblastoma. J Clin Invest.

[50] Korsmeyer SJ, Shutter JR, Veis DJ, Merry DE, Oltvai ZN (1993). Bcl-2/Bax: a rheostat that regulates an anti-oxidant pathway and cell death. Semin Cancer Biol.

[51] Yang W, Wu PF, Ma JX, Liao MJ, Wang XH, Xu LS (2019). Sortilin promotes glioblastoma invasion and mesenchymal transition through GSK-3β/β-catenin/twist pathway. Cell Death Dis.

[52] Lee Y, Lee JK, Ahn SH, Lee J, Nam DH (2016). WNT signaling in glioblastoma and therapeutic opportunities. Lab Invest.

[53] Chen Y, Fang R, Yue C, Chang G, Li P, Guo Q (2020). Wnt-induced stabilization of KDM4C is required for Wnt/β-catenin target gene expression and glioblastoma tumorigenesis. Cancer Res.

[54] Zhang K, Zhang J, Han L, Pu P, Kang C (2012). Wnt/beta-catenin signaling in glioma. J Neuroimmune Pharmacol.

[55] Tomar VS, Patil V, Somasundaram K (2020). Temozolomide induces activation of Wnt/β-catenin signaling in glioma cells via PI3K/Akt pathway: implications in glioma therapy. Cell Biol Toxicol.

[56] Du L, Wang C, Meng L, Cheng Q, Zhou J, Wang X (2018). The study of relationships between pKa value and siRNA delivery efficiency based on tri-block copolymers. Biomaterials.

[57] Karamian A, Nazarian H, Ziai SA, Zarnani AH, Salehpour S, Paktinat S (2020). Pyrvinium pamoate inhibits proliferation and invasion of human endometriotic stromal cells. Hum Exp Toxicol.

[58] Wu G, He X (2006). Threonine 41 in beta-catenin serves as a key phosphorylation relay residue in beta-catenin degradation. Biochemistry.

[59] Sokolosky M, Chappell WH, Stadelman K, Abrams SL, Davis NM, Steelman LS (2014). Inhibition of GSK-3beta activity can result in drug and hormonal resistance and alter sensitivity to targeted therapy in MCF-7 breast cancer cells. Cell Cycle.

[60] Majewska E, Szeliga M (2017). AKT/GSK3beta signaling in glioblastoma. Neurochem Res.

[61] Valenta T, Hausmann G, Basler K (2012). The many faces and functions of beta-catenin. EMBO J.

[62] Yong X, Tang B, Xiao YF, Xie R, Qin Y, Luo G (2016). Helicobacter pylori upregulates Nanog and Oct4 via Wnt/β-catenin signaling pathway to promote cancer stem cell-like properties in human gastric cancer. Cancer Lett.

[63] Chou TC (2010). Drug combination studies and their synergy quantification using the Chou-Talalay method. Cancer Res.

[64] Di Veroli GY, Fornari C, Wang D, Mollard S, Bramhall JL, Richards FM (2016). Combenefit: an interactive platform for the analysis and visualization of drug combinations. Bioinformatics.

