# Supplementary figures and images for: Pyrvinium pamoate regulates MGMT expression through suppressing the Wnt/β-catenin signaling pathway to enhance the glioblastoma sensitivity to temozolomide
Source: Cell Death Discov. 2021 Oct 12;7:288. doi: 10.1038/s41420-021-00654-2 (PMC8511032; doi:10.1038/s41420-021-00654-2)

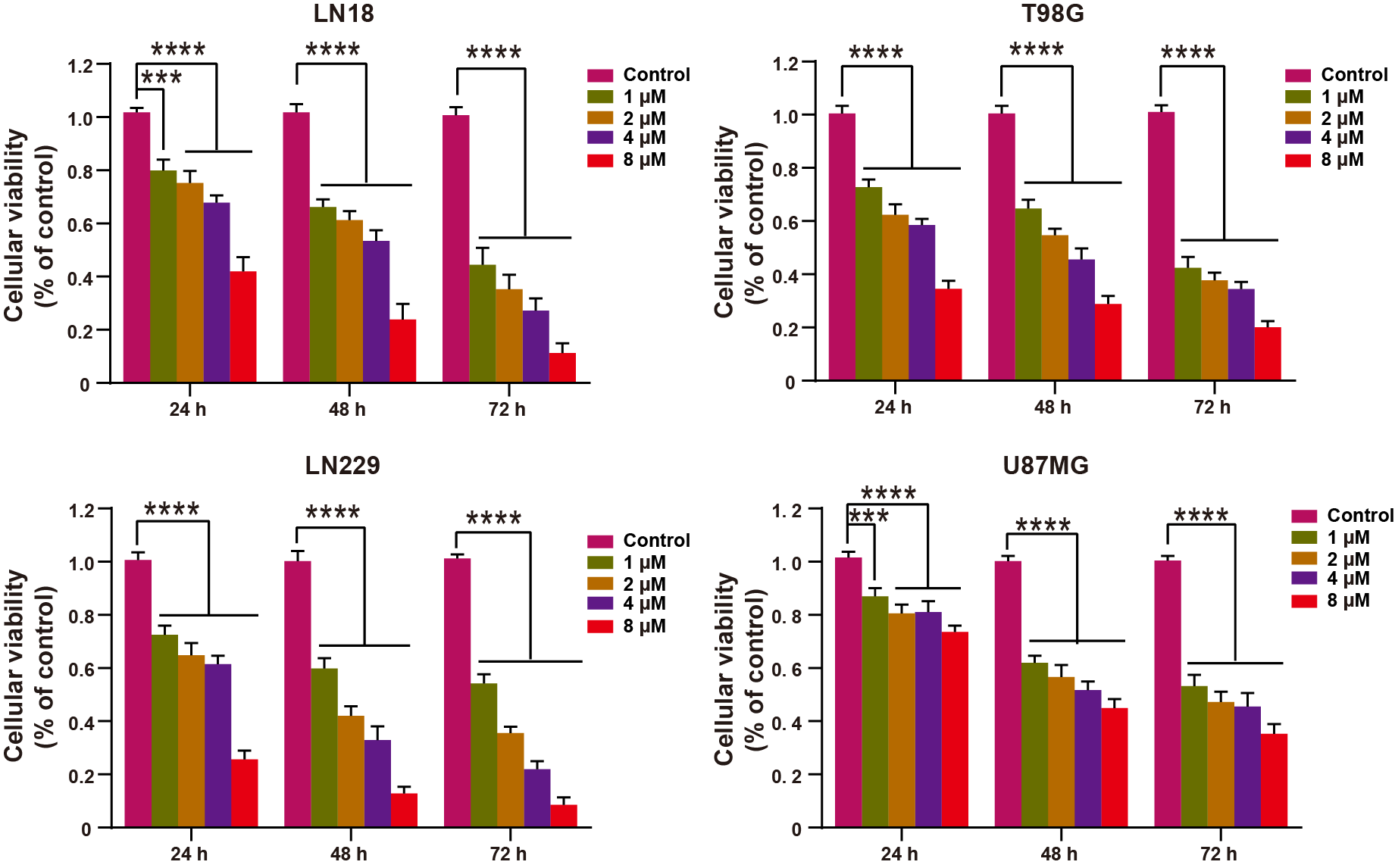

Supplement: Supplementary file 1 — Figure S1 [file 41420_2021_654_MOESM1_ESM.png]

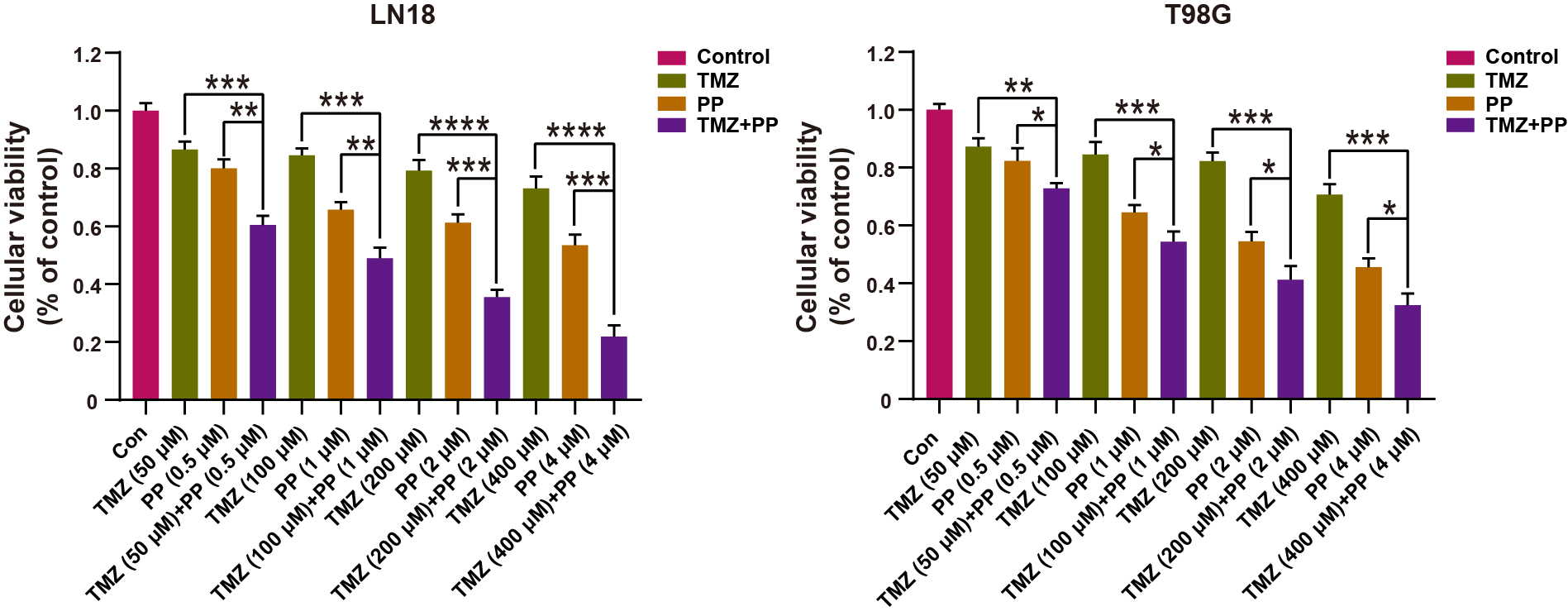

Supplement: Supplementary file 2 — Figure S2 [file 41420_2021_654_MOESM2_ESM.png]

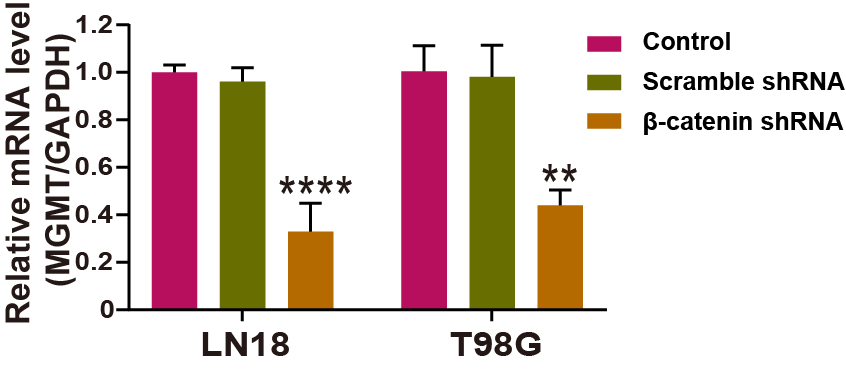

Supplement: Supplementary file 3 — Figure S3 [file 41420_2021_654_MOESM3_ESM.png]

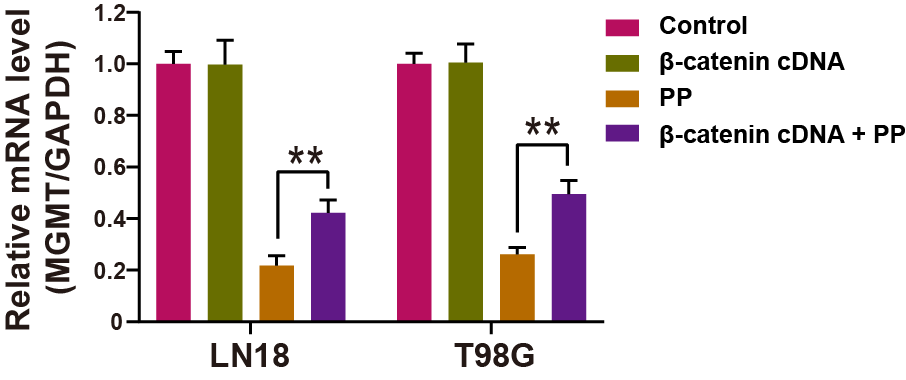

Supplement: Supplementary file 4 — Figure S4 [file 41420_2021_654_MOESM4_ESM.png]
