# Supplementary material for: Pyrvinium pamoate regulates MGMT expression through suppressing the Wnt/β-catenin signaling pathway to enhance the glioblastoma sensitivity to temozolomide
Source: Cell Death Discov. 2021 Oct 12;7:288. doi: 10.1038/s41420-021-00654-2 (PMC8511032; doi:10.1038/s41420-021-00654-2)
Supplement: Supplementary file 5 — Supplementary information [file 41420_2021_654_MOESM5_ESM.docx]

**Supplemental Information**

**Figure legend**

**Fig. S1** MTT assay showed that PP inhibited the viabilities of LN18, T98G, LN229 and U87MG cells in a dosage dependent manner. Data were presented in the manner of mean ± SD (*n* = 3). ***, *p* < 0.005; ****, *p* < 0.001.

**Fig. S2** LN18 and T98G cell viability under PP, TMZ, and TMZ+PP treatment at different contents. Data were presented in the manner of mean ± SD (*n* = 3). *, *p* < 0.05; **, *p* < 0.01; ***, *p* < 0.005; ****, *p* < 0.001.

**Fig. S3** β-catenin knockdown reduced the mRNA levels of MGMT within LN18 and T98G cells, as verified by qRT-PCR (*n* = 3). **, *p* < 0.01; ****, *p* < 0.001.

**Fig. S4** β-catenin over-expression partially restored PP-caused reduced mRNA level of MGMT within LN18 and T98G cell lines, as evidenced by qRT-PCR (*n* = 3). **, *p* < 0.01.
